# Supplementary material for: Pectin methylesterase selectively softens the onion epidermal wall yet reduces acid-induced creep
Source: J Exp Bot. 2020 Feb 1;71(9):2629–40. doi: 10.1093/jxb/eraa059 (PMC7210771; doi:10.1093/jxb/eraa059)

Supplemental data for:

Pectin methylesterase selectively softens the onion epidermal wall yet reduces acid-induced creep

by Xuan Wang, Liza Wilson, and Daniel J. Cosgrove

Department of Biology, 208 Mueller Lab, Pennsylvania State University, University Park, PA 16802 USA

*Corresponding author: email dcosgrove@psu.edu

Table S1: Effect of Ca on indentation modulus of untreated onion walls
Table S2: Effect of Ca on indentation modulus of PME-pretreated onion walls
Table S3: Effect of Mg on indentation modulus of untreated onion walls
Table S4: Effect of Mg on indentation modulus of PME-pretreated onion walls

Fig. S1: Effects of PME and BSA on onion wall surface roughness.

Supplementary Tables S1-4: Effects of 100 mM $CaCl_2$ and $MgCl_2$ on indentation modulus of onion epidermal walls with and without PME pretreatment.
Values related to the cation effects are highlighted. Analysis of variance was used to factor out variablity due to the cell wall sample.
For each treatment, ten indentations were measured in three cells.

Table S1

Effect of Ca on indentation modulus of untreated onion walls

Anova: Two-Factor With Replication

	**Treatment:** control	CaCl2	Total	RATIO	
cell2					
Count	10	10	20		
Sum	5.72	7.00	12.72		
Average	0.57	0.70	0.64	1.22	
Variance	0.0030	0.0456	0.0274		
cell3					
Count	10	10	20		
Sum	7.51	19.80	27.32		
Average	0.75	1.98	1.37	2.64	
Variance	0.0056	1.4077	1.0669		
cell6					
Count	10	10	20		
Sum	11.67	25.72	37.39		
Average	1.17	2.57	1.87	2.20	
Variance	0.0397	0.7301	0.8844		
Total					
Count	30	30			
Sum	24.897	52.525			
Average	0.830	1.751		2.11	significant
Variance	0.079	1.309			

ANOVA

Source of Variation	*SS*	*df*	*MS*	*F*	*P-value*	*F crit*
cell	15.38391	2	7.691957	20.67998	2.15E-07	3.168246
treatment	12.72177	1	12.72177	34.20274	2.98E-07	4.019541
Interaction	4.788611	2	2.394305	6.437137	0.003109	3.168246
Within	20.0854	54	0.371952			
Total	52.9797	59				

Table S2

Effect of Ca on indentation modulus of PME-pretreated onion walls

Anova: Two-Factor With Replication

	**Treatment:** PME	CaCl2	Total	RATIO	
cell3					
Count	10	10	20		
Sum	5.421	20.870	26.291		
Average	0.542	2.087	1.315	3.85	
Variance	0.023	0.273	0.768		
cell4					
Count	10	10	20		
Sum	4.914	30.360	35.274		
Average	0.491	3.036	1.764	6.18	
Variance	0.018	0.936	2.156		
cell5					
Count	10	10	20		
Sum	2.271	11.921	14.192		
Average	0.227	1.192	0.710	5.25	
Variance	0.006	0.475	0.473		
Total					
Count	30	30			
Sum	12.61	63.15			
Average	0.42	2.11		5.01	significant
Variance	0.03	1.11			

ANOVA

Source of Variation	*SS*	*df*	*MS*	*F*	*P-value*	*F crit*
cell	11.19218	2	5.59609	19.39396	4.49E-07	3.168246
treatment	42.57995	1	42.57995	147.5662	4.51E-17	4.019541
Interaction	6.3847	2	3.19235	11.06349	9.4E-05	3.168246
Within	15.5816	54	0.288548			
Total	75.73843	59				

Table S3

Effect of Mg on indentation modulus of untreated onion walls

Anova: Two-Factor With Replication

	**Treatment:** control	MgCl2	Total	Ratio	
cell9					
Count	10	10	20		
Sum	16.96	19.28	36.25		
Average	1.70	1.93	1.81	1.14	
Variance	0.156	0.523	0.336		
cell14					
Count	10	10	20		
Sum	12.23	16.85	29.08		
Average	1.22	1.69	1.45	1.38	
Variance	0.084	0.322	0.249		
cell15					
Count	10	10	20		
Sum	23.24	19.42	42.67		
Average	2.32	1.94	2.13	0.84	
Variance	4.306	0.799	2.457		
Total					
Count	30	30			
Sum	52.43	55.56			
Average	1.75	1.85		1.06	no significant difference
Variance	1.62	0.52			

ANOVA

Source of Variation	*SS*	*df*	*MS*	*F*	*P-value*	*F crit*
cell	4.617734	2	2.308867	2.237719	0.116506	3.168246
treatment	0.163261	1	0.163261	0.15823	0.69236	4.019541
Interaction	1.905127	2	0.952563	0.92321	0.40342	3.168246
Within	55.71692	54	1.031795			
Total	62.40305	59				

Table S4

Effect of Mg on indentation modulus of PME-pretreated onion walls

Anova: Two-Factor With Replication

	**Treatment:** PME	PME MgCl2	Total	RATIO	
cell1					
Count	10	10	20		
Sum	6.801	13.293	20.094		
Average	0.680	1.329	1.005	1.95	
Variance	0.126	1.347	0.808		
cell9					
Count	10	10	20		
Sum	4.70	18.91	23.60		
Average	0.47	1.89	1.18	4.02	
Variance	0.02	1.34	1.17		
cell11					
Count	10	10	20		
Sum	7.09	20.24	27.32		
Average	0.71	2.02	1.37	2.85	
Variance	0.01	3.61	2.17		
Total					
Count	30	30			
Sum	18.587	52.434			
Average	0.620	1.748		2.82	significant
Variance	0.062	2.045			

ANOVA

Source of Variation	*SS*	*df*	*MS*	*F*	*P-value*	*F crit*
cell	1.306897	2	0.653448	0.607958	0.548146	3.168246
treatment	19.09388	1	19.09388	17.76464	9.57E-05	4.019541
Interaction	1.749094	2	0.874547	0.813665	0.44859	3.168246
Within	58.04056	54	1.074825			
Total	80.19043	59				

Fig. S1. Effects of PME and BSA on onion wall surface roughness.
(A) AFM height images of onion epidermal cell wall surface scanned in HEPES buffer. (A') AFM images of the same cell wall sample scanned after PME treatment. Scale bar = 400 nm. (B) Roughness measurements of wall surface ± PME (n=6). Statistical significance was determined by Student's t-test (paired, ** $p < 0.01$). (C) AFM height image of onion epidermal cell wall surface scanned in HEPES buffer. (C') Corresponding AFM image of the same cell wall sample scanned with BSA in the buffer. Scale bar = 400 nm. (D) roughness measurements of wall surface ± BSA (n = 3). Statistical significance was determined by Student's t-test (paired, ** $p < 0.01$).

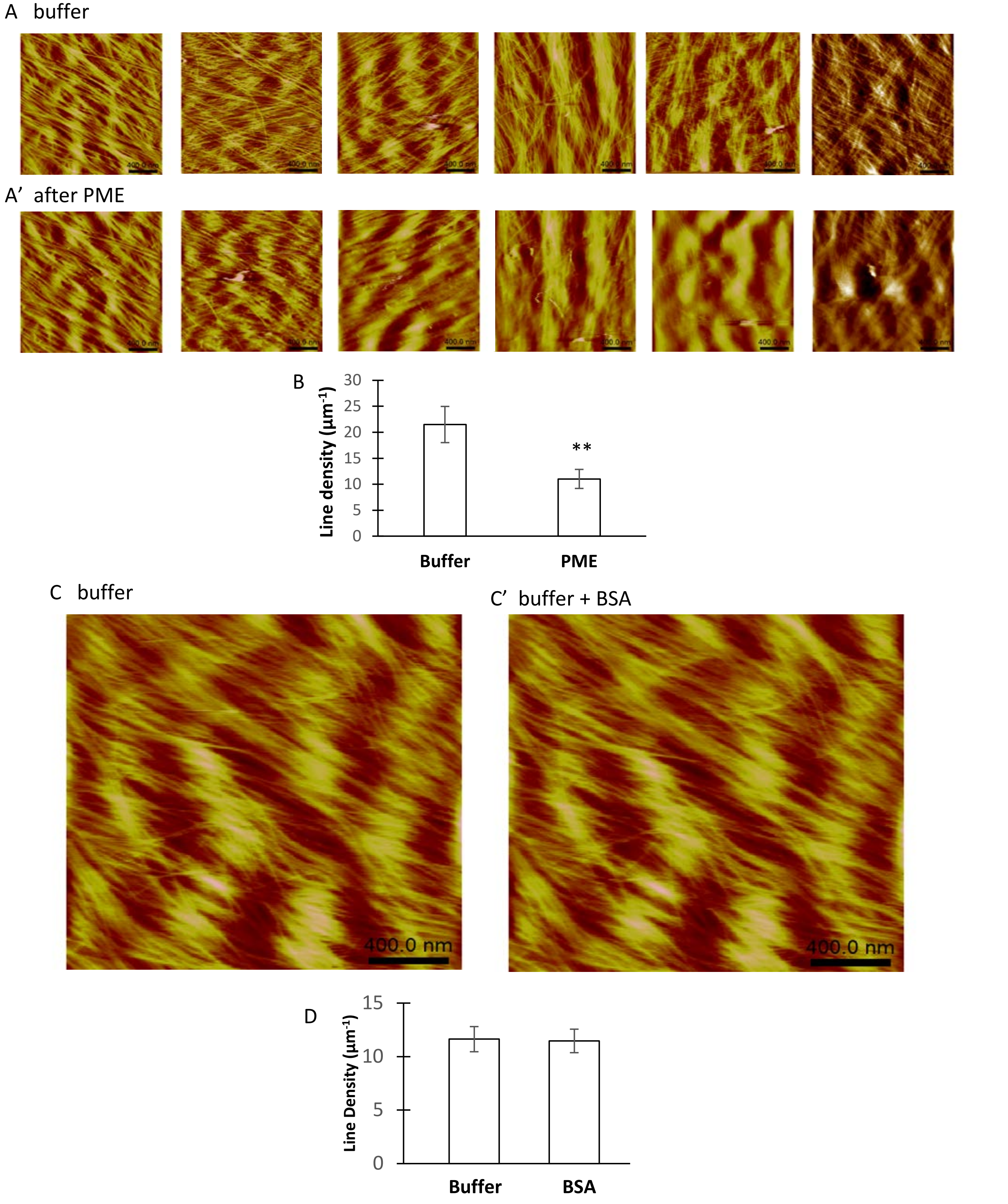

Supplement: eraa059_suppl_Supplementary_Tables_S1-S4_Figure_S1 [file eraa059_suppl_supplementary_tables_s1-s4_figure_s1.pdf]
